# Multimodal Treatment of Patients with Mental Symptom Load: A Pre–Post Comparison

**DOI:** 10.3390/jcm8101610

**Published:** 2019-10-03

**Authors:** Dieter Melchart, Volker Fischer, Jingzhang Dai, Stefan Hager, Lisa Dersch, Beatrice E. Bachmeier

**Affiliations:** 1 Competence Centre for Complementary Medicine and Naturopathy (CoCoNat), Technical University of Munich, 80801 Munich, Germany; Dieter.melchart@tum.de (D.M.); lisa.dersch@tum.de (L.D.); 2 Institute for Complementary and Integrative Medicine, University Hospital Zurich, University of Zurich, CH1903 Zurich, Switzerland; 3 TCM Hospital, 93444 Bad Kötzting, Germany; v.fischer@tcm.info (V.F.); jingzhangdai@hotmail.com (J.D.); s.hager@tcm.info (S.H.); 4 Beijing University of Chinese Medicine, Beijing 100105, China; 5 Institute of Laboratory Medicine, University Hospital, LMU Munich, 81377 Munich, Germany

**Keywords:** mental symptom load, psychosomatic disorders, multimodal treatment, ICD-10 symptom rating (ISR), Traditional Chinese Medicine (TCM), prospective observational study

## Abstract

The Traditional Chinese Medicine (TCM) Hospital in Bad Kötzting, Germany, is treating chronically ill patients, covering a broad range of indications. The aim of this study was to prove the efficacy of a multimodal intervention combining mainstream medicine with TCM treatments on the severity of psychopathological symptoms. Out of 966 patients with chronic psychosomatic disease treated 2017 at the TCM Hospital, we selected 759 patients according to specific criteria and analyzed the outcomes after multimodal intervention. The patients completed a validated questionnaire (International Statistical Classification of Diseases (ICD) Symptom-Rating-(ISR)) at admission, discharge, and follow-up. The most frequent ICD-10 diagnoses were “diseases of the musculoskeletal system and connective tissue” (28.5%), “mental and behavioral disorders” (23.7%), and “diseases of the nervous system” (13.8%). Regarding ISR symptom load, “depressive syndrome” and “anxiety syndrome” were the leading burdens showing remissions of about 40%–60% with moderate (0.588) to strong (1.115) effect sizes (Cohen’s *d*) after treatment. ISR total scores at discharge and follow-up were remarkably lower after intervention (0.64 and 0.75, respectively) compared to 1.02 at admission with moderate to strong effect sizes (0.512–0.815). These findings indicate a clinically relevant relief from mental symptom load after intervention with lasting clinical effects for at least six months.

## 1. Introduction

The current view in conventional medicine often omits the assessment of psychosocial factors that potentially influence individual vulnerability to illness, especially concerning the development and the course of chronic diseases [1]. Psychosocial variables, like interpersonal relationships, stress, personal history, family, and economic and intellectual states, can affect illness and may be crucial in treatment success of patients with unexplained somatic symptoms [2]. Health and disease can be viewed as the result of interacting mechanisms at the cellular, tissue, organismic, interpersonal, and environmental levels [3,4]. In this context, psychosomatic medicine has evolved as a wide interdisciplinary field that is concerned with the interaction of biological, psychological, and social factors in regulating the balance between health and disease [1,5,6,7,8].

The American Psychiatric Association published in their DSM-5 (Diagnostic and Statistical Manual of Mental Disorders-5) the current rates of psychosomatic disorder in the United States, which are estimated between four and six percent [9].

Psychosomatic disorders have a high degree of chronicity. An estimated 20%–30% of all patients with major depressive episodes develop chronic disease with a medium duration of over 20 years [10,11,12]. Although psychosomatic disorders are difficult to treat or cure, symptom loads perceived by the patients should be attempted to be reduced. Therefore, different concepts of therapeutic practices and the combination of traditional and conventional, or western, medicine are becoming widely used in pain management for relief from side effects and symptoms in cancer treatment, as well as for psychosomatic disorders [13]. Many of these methods are either administered or taught by practitioners specialized in mind and body practices like, e.g., acupuncture, herbs, Tuina, and Shiatsu massage. The most frequently used application in the field of complementary medicine is Traditional Chinese Medicine (TCM), a holistic approach to health, intended to harmonize body, mind, and spirit. The principles of TCM are based upon the theory of harmony of two opposite forces, named Yin and Yang. All diagnostic and therapeutic TCM procedures focus on that feature, which is similar to the homeostatic state in western medicine. A key aspect of TCM practice is the use of herbal treatments. The need for additional evidence-based research in the context of efficacy and safety of TCM procedures is urgent. In particular, the combination of TCM and conventional medicine might cause herb–drug interactions. According to the National Center for Complementary and Integrative Health of the National Institute of Health (NIH), the term “complementary” is always used as “together with” and not “in place of” conventional medicine [14].

Due to the individual symptoms and signs of psychosomatic patients, tools to evaluate and measure distress, well-being, and symptom load are highly valuable in psychosomatic medicine. The method of patient-reported outcomes (PROs), which includes any report directly from patients about how they feel in relation to a therapy, is well established as a psychosomatic tool [15,16]. The (International Statistical Classification of Diseases) ICD-10 Symptom Rating (ISR), is an economic self-assessment instrument regarding both resources and time and can be employed to record psychological symptoms and to standardize ICD-10 diagnosis [17].

In our study, we used the ISR as tool to describe the symptom load and to rate the success of multimodal intervention in a population of 966 patients with chronic mental disorder [18,19]. The TCM Hospital in Bad Kötzting, Germany, has experience in the treatment of psychosomatic disorders for over 27 years and is part of a well-established network that ranges from Bavarian Health resorts to the Beijing University of Chinese Medicine. In the context of this network, patients with psychosomatic disorders, most of them with a high degree of chronicity, are treated according to a combination of psychosomatic medicine, TCM, and lifestyle medicine. The psychoeducational intervention has been developed by Melchart and colleagues (one of the authors of this paper) in the last 25 years from a strictly TCM approach to a multimodal combination of TCM, psychosomatic medicine, and lifestyle medicine. The patients thereby benefit from TCM diagnostics, TCM remedies, acupuncture, Tuina and QiGong in combination with western diagnostics, pharmacotherapy, and consultations as needed. Additionally, patients receive diagnostics and individual therapies as part of psychosomatic medicine and psychoeducation, life style instructions, and Yangsheng from lifestyle medicine [20]. This multimodal approach has proven to be successful [20,21], especially for the treatment of unspecific symptoms, which are perceived subjectively by the individual and negatively impact their every-day lives.

As the numbers of patients with chronic psychosomatic disorders are continuously rising, in part due to increasing numbers of people suffering from distress, burnout, and tedium, general practitioners are unable to manage the increase in patients with psychosomatic symptoms. The use of psychotropic drugs in combination with analgesics has not proven to be successful, yet causes long-term adverse events [22]. In this context, the multimodal treatment approach of the TCM Hospital represents an interesting and promising alternative.

To evaluate the efficacy of this multimodal treatment approach, we selected 759 patients out of a collective of 966 patients with chronic psychosomatic disorders to analyze changes in ISR symptom loads between admission in the TCM Hospital and discharge as well as follow-up.

## 2. Methods

### 2.1. TCM Hospital

The TCM Hospital Bad Kötzting is historically the first inpatient facility in Germany with an emphasis on TCM as the main treatment modality. It is a government-licensed 75-bed hospital, which provides care for about 1000 inpatients and more than 2000 outpatients a year. Over 90% of the inpatients are fully covered by the statutory health insurance; about 10% are self-paying patients. In most cases, the hospital stay is about 4 weeks.

Over the past 27 years, the treatment concept has developed from a strictly TCM approach to a multimodal combination of TCM, psychosomatic medicine, and lifestyle medicine [21]. The majority of our patients present with clinical problems with a high degree of chronicity and complexity. They are frequently transcribed to us with diagnoses of neurological or orthopedic diseases, or from internal medicine domains. Most patients present after years of conventional treatment that has proven unsatisfactory. Essentially, our specialty is the multimodal treatment of somatic complaints in conjunction with acute psychological or psychiatric comorbidity. Thus, the vast majority of our patients carry both somatic and psychiatric diagnoses.

### 2.2. Documentation System

The data analysis of this outcome study was based on an ongoing comprehensive documentation system called VITERIO [15]. Briefly, VITERIO is a web-based health portal useful in clinical practice for assessing response to interventions. All clinical data from one patient can be collected and stored centrally, so are always available for the patient as well as clinicians and health professionals, if a written consent is provided.

Both at admission and discharge, all in-patients participating in our outcome study filled an approved questionnaire (ICD-Symptom-Rating-ISR). The questionnaires were deposited electronically in VITERIO along with the corresponding ICD-10 diagnosis and all other associated clinical and health patient data.

### 2.3. ICD-10 Diagnosis

Possible psychological disorders were classified for 966 patients at admission at the TCM Hospital Bad Kötzting according to the international classification of diseases (ICD-10). Only one diagnosis was allowed.

### 2.4. ISR (ICD-10-Symptom-Rating)

The ISR questionnaire developed by Tritt et al. was used for the assessment of presence as well as severity of psychological symptoms and consists of 29 items [18]. It is based on chapter F of the German ICD classification system ICD-10-GM [23]. Each item is rated on a 5-point Likert scale: = does not apply, 1 = a little, 2 = quite a bit, 3 = to a great extent, and = extremely.

The ISR total score was calculated based on the five mentioned answer categories. The total score five sub-scores are defined each covered by standardized items: depressive syndrome (4 items), anxiety syndrome (4 items), compulsive-obsessive syndrome (3 items), somatoform syndrome (3 items), and eating disorder syndrome (3 items).

### 2.5. Study Design

**Figure 1:**
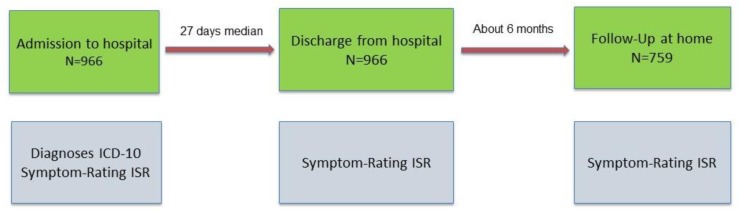
Study design and number of patients. ICD stands for International Statistical Classification of Diseases. ISR stands for ICD Symptom Rating.

We performed an observational study to evaluate the effect of multimodal treatment on the symptom loads of patients with chronic psychosomatic disorders. The study was performed according to the STROBE guidelines, which is a checklist providing guidance for appropriately reporting observational research [24,25]. At admission, all inpatients were diagnosed according to the western routine and diseases were coded according to ICD-10. Psychological disorders at admission and discharge from the TCM Hospital as well as follow-up were symptomatically evaluated by the valid, standardized, and patient-reported questionnaire ISR (ICD-10 symptom rating) (Figure 1). The timeframe of admission was between 21 January and 29 December 2017. Timeframe of discharge was between the 20 January 2017 and the 30 January 2018. Timeframe of follow-up was between 17 July 2017 and the 30 August 2018.

Research Involving Human Subjects. Ethics approval: All scientific activities are authorized and reviewed by an Academic Exchange Agreement between the Bejing University of Chinese Medicine and Technische Universität München. Informed consent: The patients gave their written consent on admission to the TCM Klinik Bad Kötzting.

Values at admission were compared with values at discharge and follow-up and statistical analysis was performed to evaluate the effect of the multimodal intervention.

### 2.6. Patient Admission

Before admission patients filled out questionnaires detailing their physical and psychological complaints, medical history, desired treatment outcome, previous and current diagnoses, and findings. Patients completed Internet-based questionnaires in the web-based health portal VITERIO to facilitate assessment of various areas of health, stress, coping, resources, and satisfaction with multiple domains of life. Data were considered the source of information in the course of the initial medical and psychological assessments.

After having received written consent, a physician trained in western medicine examined the patients. As part of this routine, the patients underwent a first comprehensive interview. The physician presented the findings from this interview to a TCM doctor (mediated by a translator) as well as the assigned psychotherapist in the presence of the patient. The TCM doctor built a TCM diagnoses based on the medical history, symptoms, clarifying questions, as well as a tongue and pulse examination, and prescribed suitable TCM treatments. The psychotherapist gathered information about the medical background of the patient to first establish a psychological rapport.

### 2.7. Multimodal Intervention Strategy

Above all, we tried to convey a psychosomatic understanding of the mind–body connection. This often necessitates changing the patient’s implicit ‘disease model’ from being largely external to more internal. Our therapy concept is based on a combination of western psychological insights, health and lifestyle information, and eastern mind–body concepts. Therefore, we educate our patients about the basics of TCM, particularly that disturbances in the flow of Qi due to internal factors, such as excessive or imbalanced emotions, can lead to the manifestation of functional (Qi-flow disturbances) or somatic symptoms (Qi- and blood-flow disturbances).

#### 2.7.1. TCM Intervention

The TCM diagnoses and treatments were performed by doctors and professors of the Beijing University of Chinese Medicine and its associated university hospitals. The primary TCM treatment modalities included highly concentrated Chinese medicinal remedies, acupuncture, Tui Na (massage), and Qigong. All treatments and interventions were designed to address the observed patterns of symptoms. Chinese medicinal remedies (in form of decoction) had to be taken by the patients twice daily. Most patients received three acupuncture treatments per week. Since a vast majority of patients also suffered from pain in their muscles, joints, or back, two manual treatments—Either Tui Na massage or individual Qigong treatment—Had to be added to the prescribed regimen in most cases.

All patients participated in a series of 18 exercises derived from time-tested Tai Chi-Qigong traditions. The exercises comprise slow movements, deep breathing techniques, and imagery components. These meditative movements were practiced in groups for 30 min in the morning and in the afternoon. Video instructions were also accessible on demand for all patients. All patients were introduced to the traditional chanting of healing sounds as well as a self-care program called ‘3-1-2’. Those numbers stand for three acupressure points, deep diaphragmatic breathing, and two squats. Healing sounds and 3-1-2 were taught as self-care interventions. All patients received nutritional counselling, which provided general recommendations in accordance with TCM dietary principles as well as specific guidance tailored to the individual’s TCM diagnoses.

The patients were encouraged to practice a series of 18 exercises derived from the Tai Chi-Qigong traditions (see above) after discharge twice daily. The patients were instructed how to optimize their diet at home according to Chinese dietary principles. TCM medicinal remedies should be continued at a lower dosage for several months. The patients were advised to incorporate the Tai Chi-Qigong exercises as well as numerous other dietary and lifestyle recommendations into their daily routine.

#### 2.7.2. Psychotherapeutic and Psychoeducational Intervention

The conceptual framework of the psychological treatment components was behavioral in orientation with an emphasis on (a) improving the regulation of affects, (b) reducing dysfunctional cognitions, and (c) teaching patients to take charge of their healing process through lifestyle adjustments, self-care applications, and preventive measures.

In detail, a unique feature of our treatment approach is the use of a comprehensive, web-based health-maintenance program [15,26]. Even prior to admission, patients receive access to this program and are encouraged to take advantage of its many features and components. The data entered by patients provide information for our therapists; simultaneously, they serve as a progress assessment tool for the patients themselves. The data entered by the hospital include diagnostic information (including laboratory data) and a record of all therapeutic interventions. Thus, the patient has access to self-generated as well as hospital-generated information. The health maintenance program is linked to abundant in-depth information, offering each patient the opportunity to further explore many health and lifestyle topics. The patients receive a professional introduction and practical lessons through multi-disciplinary presentations and lectures [26]. The health maintenance program helps patients to receive information and become actively engaged in their healing process, both during and after the inpatient stay.

### 2.8. Statistical Analysis

Social-demographic data, such as year of birth, sex, ICD-10-diagnoses, and the ICD-10-Symptom-Rating (ISR), were collected through the web-based tool VITERIO [15]. ISR data were collected at admission, discharge, and 6 months after discharge (follow-up).

The principal outcome variable was a reduction in ISR total score at discharge and follow-up. Secondary outcome variables were decreased values in the five ISR sub-scores: depressive syndrome, anxiety syndrome, compulsive-obsessive syndrome, somatoform syndrome, and eating disorder syndrome. Data entry screens were used to revise incorrect entries (i.e., time errors).

Quantitative data are expressed as the mean ± standard deviation. The paired *t*-test was used to determine the mean differences between data at admission and data at discharge and data at follow-up. The reported *p*-values are two-tailed with level of significance set at 0.05. Cohen’s *d* effect sizes were calculated using a random effects model [27,28,29].

To reduce the standard error of the difference between the means Cohen’s *d* was computed by the following formula:

(1) $$d=t\times \sqrt{\frac{2\times \left(1-\;r\right)}{N}}$$

where *r* is the correlation across pairs of measures and *t* is the test statistic calculated by dividing the mean of differences by the standard error of differences [30]. *d* is interpreted following Cohen’s description [28].

#### Data Selection

The data at admission were maximum of 61 days before admission and at the least of 7 days after admission. The data at discharge included a maximum of 7 days before discharge and not later than the day of discharge. Data at follow-up included minimum of 154 days to a maximum of 300 days after discharge.

Statistical analysis were performed using IBM SPSS Statistics Version 25.0 (Armonk, New York, NY, USA).

We also applied the baseline observation carried forward (BOCF) technique to investigate the stability of the results. BOCF is based on the assumption that at the timepoint of follow-up (6 months after discharge from the TCM Hospital), the patient’s sustained benefit from multimodal intervention is not inferior to the initial ISR value at baseline. Hence, we imputed missing data from follow-up with the scores from baseline (admission).

## 3. Results

### 3.1. Sample Description

We determined psychological disorders according to the ICD-10 diagnosis in a sample of 966 in-patients of the TCM Hospital in Bad Kötzting, Germany, at admission and discharge, who had a history of chronic psychosomatic disease with median duration of seven years, and were treated between 2 January 2017 and 30 January 2018 (exact timeframes are provided in Section 2).

**Figure 2:**
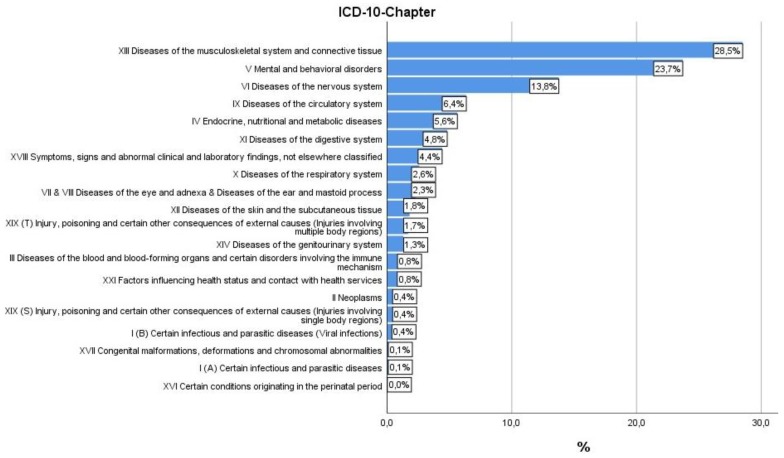
ICD-10 diagnosis of patients at discharge.

Within the sample of 966 patients, the most frequent ICD-10 diagnoses were “Diseases of the musculoskeletal system and connective tissue” (28.5%), “Mental and behavioral disorders” (23.7%), and “Diseases of the nervous system” (13.8%) (Figure 2).

ICD-10 symptom loads according to ICD-10 symptom rating (ISR) were determined at admission (*n* = 966), discharge (*n* = 966), and follow-up (*n* = 759) (Table 1).

Of the 966 patients who were included into the study, we identified 759 eligible for our outcome analysis by filtering according to the following criteria:

1. Patients who had an ISR diagnosis at admission;
2. Valid ISR values were available for all three time points (admission, discharge, and follow up);
3. Patients were stationary at the TCM Hospital Bad Kötzting for a minimum of 14 and a maximum of 49 days;
4. Patients had a follow-up between 180 and 300 days after discharge from the hospital.

This collective of 759 patients was used for our outcome study. The mean age of this sample was 55.74 ± 11.55 years and 76.2% were women (Table 1).

### 3.2. Symptom Load (ISR Total Score and Subscores) at Admission, Discharge, and Follow-Up

**Figure 3:**
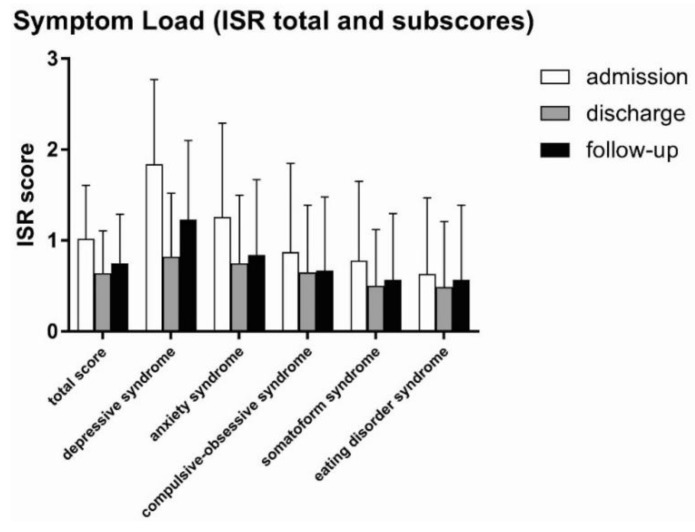
Symptom loads (ISR total score and sub-scores) at admission, discharge, and follow-up. Patients undergoing multimodal intervention at the Traditional Chinese Medicine (TCM) Hospital had remarkably lower symptom load regarding ISR total score and the sub-scores “depressive syndrome” and “anxiety syndrome”. This positive and beneficial effect was also observed for the sub-scores “compulsive-obsessive syndrome”, “somatoform syndrome”, and “eating disorder syndrome”, but to a lesser degree.

To evaluate the effect of our multimodal intervention, ISR total and sub-scores were recorded at admission, discharge, and six months after discharge (follow-up). We found significant differences in symptom load between admission and discharge/follow-up (Table 2, Figure 3). The ISR total scores at discharge and follow-up were remarkably lower after intervention with a values of 0.64 and 0.75, respectively, compared to 1.02 at admission in the TCM Hospital and had effect sizes (measured by Cohen’s *d*) of 0.815 and 0.512, corresponding to a moderate to strong effect respectively.

The best results were observed in the sub-scores “depressive syndrome” and “anxiety syndrome”. Likewise, remission of the sub-score “depressive syndrome” at discharge (0.70) was about 60% of the initial value at admission (1.84) and had an effect size as measured by Cohen’s *d* of 1.115, indicating a very strong effect.

Similarly, the sub-score “anxiety syndrome” was lower at discharge (0.75) than at admission (1.26) with an effect size of 0.588. Differences were observed in all other sub-scores with smaller effect sizes (Cohen’s *d*) of 0.225 (eating disorder syndrome), 0.253 (compulsive-obsessive syndrome), and 0.352 (somatoform syndrome). In general, the effects of our multimodal intervention were sustainable and still persistent at follow-up, however, to a minor degree (Table 2, Figure 3). Differences in ISR total score and sub-scores between admission and discharge/follow-up were statistically highly significant (paired *t*-test) with *p*-values < 0.001 (***), with a minor exception for “eating disorder” where differences between admission and follow-up had a *p*-value of < 0.5 (*).

**Figure 4:**
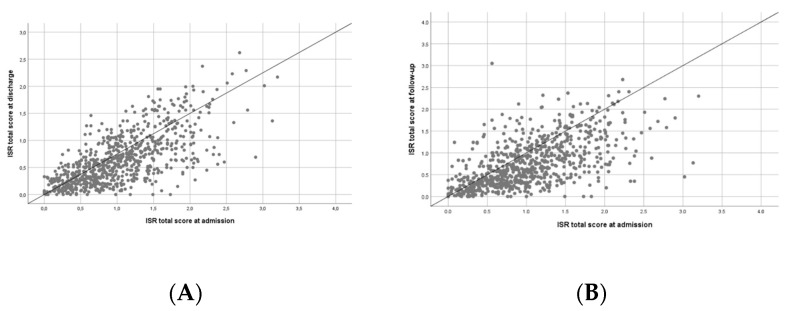
Effect of multimodal intervention. The scatter blots illustrate the effect of the multimodal intervention at (**A**) discharge from the TCM Hospital or (**B**) follow-up. Each dot represents one patient. The dots below the line represent patients who benefitted from intervention, the dots on the line represent patients with no effect, and the dots above the line represent patients with aggravation of symptoms at (**A**) discharge or (**B**) follow-up with respect to admission.

We analyzed the effects of the multimodal intervention between admission and discharge (Figure 4A) and between admission and follow-up (Figure 4B) in every single one of the 618 patients. At discharge (Figure 4A), beneficial effects were observed for 621 (81.1%) patients, no effect was observed in 11 patients (1.4%), and 127 patients (16.7%) had aggravation of symptoms. At follow-up (Figure 4B), 563 patients (74.2%) benefitted from intervention, and 190 (25%) reported aggravation of their symptoms. In six patients (0.8%), no effect was observed.

### 3.3. Percent Distribution of ISR Symptom Loads before and after TCM Intervention

**Figure 5:**
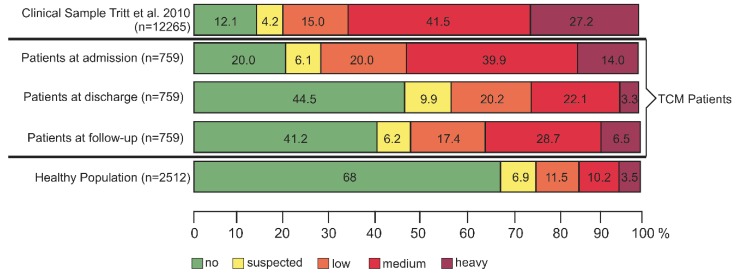
Comparison of ISR symptom loads between in-patients of the TCM Hospital Bad Kötzting, Germany, and peer samples.

We classified the symptom loads of our collective of 759 patients with complete data sets (admission, discharge, and follow-up) at admission into the five categories “no”, “suspected”, “low”, “medium”, and “heavy” and compared the loads with a clinical sample (*n* = 12,265) published by Tritt et al. [19]. We found similar distributions concerning the severity of symptom loads in both collectives (Figure 5, first two panels). In a second step, we analyzed the effect of multimodal intervention on severity of symptoms at admission, discharge, and follow-up (panels 2.3 and 4). At admission, only 20% of our patients had no symptoms according to ISR, at discharge about 44% and at follow-up 41% were symptom-free, after a median treatment duration of 27 days in the TCM Hospital (panels 2 and 3).

Benefit from stationary multimodal therapy at the TCM Hospital persisted for at least six months resulting in stable values at follow-up with 28.7% medium and 6.5% heavy symptom loads (Figure 5, panel 4).

We also analyzed changes in the single categories of symptom loads in relation to the multimodal intervention at discharge (Table 3) and at follow-up (Table 4).

Classification of symptom loads into the five categories “no”, “suspected”, “low”, “medium” and “heavy” symptom loads reveals that the share of patients who benefitted from multimodal intervention was higher (indicated in green) than the percentage of patients with aggravation of symptoms (indicated in red) both at discharge (Table 3) as well as at follow-up (Table 4).

### 3.4. Loss to Follow-Up

Out of the 966 patients who were included into the study at baseline, 759 patients had complete data sets for admission, discharge, and follow-up (see sample description in Section 2). The other 207 (21.4%) patients were declared as the loss-to-follow-up population.

Comparing the data of the 759 patients with complete datasets with the 207 patients without follow-up data, we found no significant difference in benefit from the multimodal treatment at the timepoint of discharge (Table 5).

**Figure 6:**
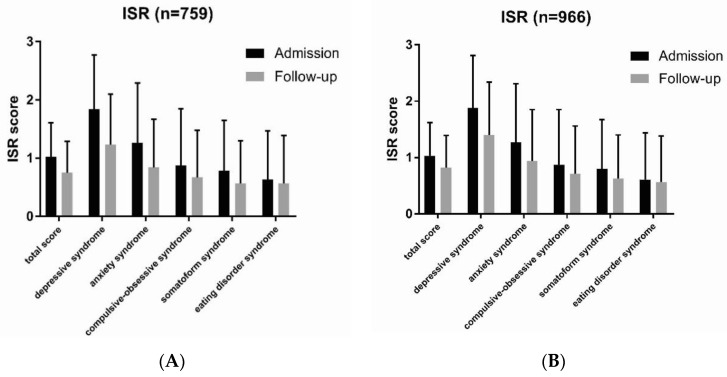
Symptom loads at admission and follow up calculated using the BOCF technique. (**A**) For patients with complete datasets (*n* = 759), improvements in symptom loads were still significant at follow-up. (**B**) For all patients (*n* = 966) and using the BOCF technique for the 207 patients lost to follow-up, the positive effect of the multimodal therapy on patients’ symptom loads was still evident with high statistical significance at follow-up.

We used the baseline observation carried forward (BOCF) technique to replace the missing follow-up data from the 207 individuals (Table 6). Our results show a significant overall improvement in ISR symptom loads regardless of any additional benefit from multimodal treatment at the timepoint of discharge for the 207 patients that were lost to follow up. Total scores as well as sub-scores (except for eating disorder syndrome) decreased with high statistical significance (Table 6), demonstrating the positive effect of multimodal therapy on ISR symptom loads (Figure 6).

## 4. Discussion

The TCM Hospital in Bad Kötzting, Germany, has specialized in the multimodal treatment of somatic complaints in combination with psychological or psychiatric comorbidity. Most of the hospitalized patients have a history of psychosomatic disease of more than seven years [31] with high degrees of chronicity and complexity. Before hospitalization, patients were treated according to conventional medical practice. The treatment concept of the TCM Hospital combines conventional and complementary treatments with elements from TCM, and psychosomatic and lifestyle medicine with a strong emphasis on TCM [20,31].

Patients in our study had the following characteristics:

1. The majority of patients suffered from a combination of mental symptoms and chronic somatic disease, mostly pain.
2. They had a long history of psychosomatic disease with high degree of chronicity.
3. Outpatient conventional therapy did not have any significant beneficial effect.
4. Patients had high symptom loads according to ISR classification.

Regarding the ISR symptom loads, patients at admission to the TCM Hospital Bad Kötzting had a similar distribution compared to a clinical sample collected from 10 German psychosomatic hospitals (*n* = 12,265 [19]) (Figure 6, upper two panels). The majority (73.9%) of our patients were suffering from their disease with more than half reporting medium (39.9%) to heavy (14%) symptoms. This justifies an intervention targeting the relief of their symptoms where, in many cases, a complete remission of the disease is unlikely. Because previous predominantly conventional treatment strategies did not lead to amelioration of symptoms in these patients with chronic psychosomatic disorders, it was appropriate to apply a combination approach of eastern TCM methods with western psychosomatic therapy approaches, which is routine in the TCM Hospital Bad Kötzting. We have published previously that TCM treatment of chronically ill patients improves significantly their quality of life [32] and that - for over 20-years patients with chronic psychosomatic disorders benefit from the unique combination between “eastern and western” treatment approaches [20,21]. An important part of the TCM treatment modalities are highly concentrated Chinese medicinal remedies, which are prescribed by the TCM doctor based on the TCM diagnosis and the observed patterns of symptoms. According to TCM doctrine, depression is caused by liver Qi stagnation; therefore, drugs that promote liver Qi circulation are applied for the treatment of depressive disorders [33]. The biomedical mechanism behind this doctrine is that Qi stagnation could hinder mitochondrial ATP synthesis in the liver and consequently disrupt the release of neurotransmitters and the transport of neurotropin to the brain [33]. The most representative prescription for the treatment of depressive disorders is Chaihu-Shugan-San, which contains seven herbs, among them Bupleuri Radix [34]. Western medicine also uses pharmacotherapy for the treatment of depressive disorders. The most common anti-depressive drugs are selective serotonin reuptake inhibitors (SSRIs), serotonin and norepinephrine reuptake inhibitors (SNRIs), atypical antidepressants, tricyclic antidepressants, and monoamine oxidase inhibitors (MAOIs). Most of them have proven side effects like weight gain, (anti-)cholinergic, gastrointestinal, endocrine, and sedating adverse events.

All patient included in our study already had a chronic psychosomatic disorder at admission to the TCM hospital and most of them received pharmacotherapy. Therefore, the combination of western psychosomatic drugs with Chinese herbal medicine can lead to undesired drug interactions [35]. Consequently, we carefully checked laboratory values corresponding to organ damage (liver and kidney) and hematology of all our patients at admission and during the course of therapy. In addition, all patients answered open questionnaires for individually perceived side effects (patient reported outcomes) in the context of our systematic complication-screening program.

These results align well with the study presented here, showing that at discharge and follow-up, patients stationary for at least 27 days in the TCM hospital had lower symptom loads, revealing the beneficial effects of the TCM intervention.

In comparison with a representative German healthy population, the symptom loads of our patients were still higher (Figure 5, lower panel). This demonstrates that the cure of chronic psychosomatic disorders remains challenging. The most frequent ICD-10 chapter diagnosis in our patient collective with complete data sets for admission, discharge, and follow-up (*N* = 759) was “Diseases of the musculoskeletal system and connective tissue” (28.5%). The reason for this is based in the history of the TCM Hospital, which was established in 1991 for the treatment of pain disorders and developed into an institution for the treatment of all kinds of psychosomatic disorders. In this context, a remarkable proportion of our patients suffered from “Mental and behavioral disorders” (23.7%).

Regarding the ISR sub-scores, values for depressive syndrome and anxiety syndrome were highest throughout all diagnoses. Considering the high prevalence of patients with somatoform pain disorders and the degree of chronicity, this is comprehensible; depressive or anxiety symptoms and somatization are strongly associated with one another [36,37,38,39]. Even young people are affected by a high prevalence of depressive symptoms and somatic complaints, which have repeatedly been found among college and university students as reported by the American College Health Association [40].

In our study, we observed the most significant improvements in patients with depressive syndromes; total scores and anxiety syndromes also showed clinically relevant effects: ISR total scores as well as the sub-scores of depressive and anxiety syndromes were lower compared with the situation at admission in the TCM Hospital with predominantly moderate to high effect sizes (Cohen’s *d*).

## 5. Conclusions

Despite the poor outcome rates regarding conventional and outpatient treatment and the high incidence of chronicity of psychosomatic disorders, we observed significant improvements at discharge and follow-up in our patients. To improve therapy benefits for patients with chronic psychosomatic disorders, we recommend a multimodal treatment consisting of TCM, and psychosomatic and lifestyle medicine with stationary sojourns during the course of therapy.

## Figures and Tables

**Table 1:** Sample description.

	*n*	Female (%)	Age (Years)	
			Mean	SD
Admission	966	74.6	55.41	11.92
Discharge	966	74.6	55.41	11.92
Follow-up (eligible for outcome analysis)	759	76.2	55.74	11.55

**Table 2:** Comparison of symptom loads between admission and discharge and admission and follow-up of 759 patients with complete data sets.

ISR	Admission		Discharge			Follow-Up		
	Mean	SD	Mean	SD	Cohen’s *d*	Mean	SD	Cohen’s *d*
Total Score	1.02	0.59	0.64	0.47	0.815 ***	0.75	0.54	0.512 ***
Sub-scores								
Depressive Syndrome	1.84	0.93	0.82	0.70	1.115 * **	1.23	0.87	0.649 ***
Anxiety Syndrome	1.26	1.03	0.75	0.75	0.588 ***	0.84	0.83	0.43 ***
Compulsive-Obsessive Syndrome	0.87	0.98	0.65	0.74	0.253 ***	0.67	0.81	0.219 ***
Somatoform Syndrome	0.78	0.87	0.50	0.62	0.352 ***	0.57	0.73	0.247 ***
Eating Disorder Syndrome	0.63	0.84	0.49	0.72	0.225 ***	0.57	0.82	0.085 *

Cohen’s *d*: >0.20 small, >0.50 moderate, >0.80 strong effect; Paired *t*-test: *** *p* < 0.001, * *p* < 0.05.

**Table 3:** Cross-classified table of the change in symptom classification between admission and discharge.

			ISR Total Discharge					Total
			No.	Suspected	Low	Medium	Heavy	
ISR total admission	no	*n*	136	12	4	0	0	152
		%	17.9%	1.6%	0.5%	0.0%	0.0%	20.0%
	suspected	*n*	32	4	7	3	0	46
		%	4.2%	0.5%	0.9%	0.4%	0.0%	6.1%
	low	*n*	85	28	23	16	0	152
		%	11.2%	3.7%	3.0%	2.1%	0.0%	20.0%
	medium	*n*	78	27	96	96	6	303
		%	10.3%	3.6%	12.6%	12.6%	0.8%	39.9%
	heavy	*n*	7	4	23	53	19	106
		%	0.9%	0.5%	3.0%	7.0%	2.5%	14.0%
total		*n*	273	338	75	153	168	25
		%	44.2%	44.5%	9.9%	20.2%	22.1%	3.3%

Note: Green indicates amelioration (*n* = 433; 57.0%), white stable symptoms (*n* = 278; 36.6%), and red aggravation (*n* = 48; 6.3%).

**Table 4:** Cross-classified table of the change in symptom classification between admission and follow-up.

			ISR Total Follow-Up					Total
			No	Suspected	Low	Medium	Heavy	
ISR total admission	no	*n*	129	4	10	9	0	152
		%	17.0%	0.5%	1.3%	1.2%	0.0%	20.0%
	suspected	*n*	29	3	7	5	2	46
		%	3.8%	0.4%	0.9%	0.7%	0.3%	6.1%
	low	*n*	72	16	29	32	3	152
		%	9.5%	2.1%	3.8%	4.2%	0.4%	20.0%
	medium	*n*	74	20	69	121	19	303
		%	9.7%	2.6%	9.1%	15.9%	2.5%	39.9%
	heavy	*n*	9	4	17	51	25	106
		%	1.2%	0.5%	2.2%	6.7%	3.3%	14.0%
total		*n*	253	313	47	132	218	49
		%	40.9%	41.2%	6.2%	17.4%	28.7%	6.5%

Note: Green indicates amelioration (*n* = 361; 47.6%), white stable symptoms (*n* = 307; 40.4%), and red aggravation (*n* = 91; 12.0%).

**Table 5:** Comparison of symptom loads between admission and discharge of 759 patients with complete data sets and of the 207 patients who were lost to follow-up.

	Complete Datasets (*n* = 759)					Loss-to-Follow-Up (*n* = 207)				
ICD-10-Symptom-Rating ISR	Admission		Discharge			Admission		Discharge		
	Mean	SD	Mean	SD	Cohen’s *d*	Mean	SD	Mean	SD	Cohen’s *d*
Total score	1.02	0.59	0.64	0.47	0.815 ***	1.07	0.61	0.73	0.49	0.645 ***
Sub-scores										
Depressive Syndrome	1.84	0.93	0.82	0.70	1.115 ***	2.03	0.93	1.07	0.82	1.041 ***
Anxiety Syndrome	1.26	1.03	0.75	0.75	0.588 ***	1.30	1.08	0.87	0.83	0.425 ***
Compulsive-Obsessive Syndrome	0.87	0.98	0.65	0.74	0.253 ***	0.85	0.99	0.67	0.76	0.193 **
Somatoform Syndrome	0.78	0.87	0.50	0.62	0.352 ***	0.85	0.88	0.57	0.68	0.339 ***
Eating Disorder Syndrome	0.63	0.84	0.49	0.72	0.225 ***	0.55	0.79	0.48	0.67	**0.113**

Cohen’s *d*: >0.20 small, >0.50 moderate, >0.80 strong effect. Paired *t*-test: *** *p* < 0.001.

**Table 6:** Comparison of symptom loads between admission and follow-up of 759 patients with complete data sets and of 966 patients including 207 patients with baseline-observation-carried-forward (BOCF).

ICD-10-Symptom-Rating ISR	Complete Datasets (*n* = 759)			Baseline-Observation-Carried- Forward (*n* = 966)		
	Admission Mean (SD)	Follow-Up Mean (SD)	Cohen’s *d*	Admission Mean (SD)	Follow-Up Mean (SD)	Cohen’s *d*
Total score	1.02 (0.59)	0.75 (0.54)	0.512 ***	1.03 (0.59)	0.82 (0.57)	0.448 ***
Sub-scores						
Depressive Syndrome	1.84 (0.93)	1.23 (0.87)	0.649 ***	1.88 (0.93)	1.40 (0.94)	0.572 ***
Anxiety Syndrome	1.26 (1.03)	0.84 (0.83)	0.43 ***	1.27 (1.04)	0.94 (0.91)	0.384 ***
Compulsive-obsessive Syndrome	0.87 (0.98)	0.67 (0.81)	0.219 ***	0.87 (0.98)	0.71 (0.85)	0.201 ***
Somatoform Syndrome	0.78 (0.87)	0.57 (0.73)	0.247 ***	0.80 (0.87)	0.63 (0.77)	0.229 ***
Eating disorder Syndrome	0.63 (0.84)	0.57 (0.82)	0.085 *	0.61 (0.83)	0.57 (0.81)	0.064 *

Note: Cohen’s *d*: >0.20 small, >0.50 moderate, >0.80 strong effect. Paired *t*-test: * *p* < 0.05, *** *p* < 0.001.
